# NADK Governs Ferroptosis Susceptibility by Orchestrating NADPH Homeostasis

**DOI:** 10.3390/antiox14121396

**Published:** 2025-11-24

**Authors:** Xinyi Chen, Yingying Zhang, Dandan Song, Fei Gui, Yuejia Cao, Yu Hong, Rong Chen, Yang Song, Chunhong Di, Jun Yang, Xiaohua Tan

**Affiliations:** 1 School of Public Health and Nursing, Hangzhou Normal University, Hangzhou 311121, China; 2 Laboratory Animal Center, Hangzhou Normal University, Hangzhou 311121, China; 3 The Affiliated Hospital, Hangzhou Normal University, Hangzhou 310015, China; chuhongdi@hanzu.edu.cn

**Keywords:** NAD kinase 1, ferroptosis, NADPH, redox homeostasis, lipid peroxidation

## Abstract

Ferroptosis, a regulated cell death pathway driven by iron-dependent lipid peroxidation, is modulated by cellular antioxidant systems, particularly the glutathione (GSH)–glutathione peroxidase 4 (GPX4) axis. NAD kinase (NADK), the only enzyme converting NAD^+^ to NADP^+^ located in cytoplasm, fuels NADPH-dependent antioxidant defenses. However, its role in ferroptosis regulation remains not fully explored. Using ferroptosis-sensitive HT1080 cells, we employed pharmacological inhibition (thioNAM), siRNA-mediated knockdown, and plasmid-driven overexpression of NADK to dissect its impact on ferroptosis. Complementary interventions with nicotinamide mononucleotide (NMN), glucose-6-phosphate dehydrogenase (G6PD) and malic enzyme 1 (ME1) were used to map metabolic interactions. Cell viability, redox metabolites (NADPH and GSH), oxidative stress markers (ROS, MDA), and protein expression were quantified. ThioNAM depleted NADP(H) and sensitized cells to RSL-3-induced ferroptosis, which was reversible with Ferrostatin-1. NADK knockdown produced similar results, reducing NADP(H) levels and amplifying lipid peroxidation. Conversely, NADK overexpression restored NADPH/GSH levels and rescued ferroptosis. NADK was essential for G6PD- and ME1-mediated NADPH production and ferroptosis resistance. Administration of ThioNAM or knockdown of NADK abolished the ferroptosis-rescuing effects of NMN, whereas NADK overexpression enhanced NMN’s ability to rescue ferroptosis by maintaining redox homeostasis. NADK is a metabolic hub in ferroptosis regulation, bridging NMN-driven NAD^+^ salvage to NADPH synthesis via G6PD/ME1. Targeting NADK offers novel strategies for diseases associated with ferroptosis.

## 1. Introduction

Ferroptosis is an iron-dependent, non-apoptotic form of regulated cell death involving widespread lipid peroxidation of cellular membranes. It is increasingly recognized as a key pathogenic mechanism underlying various pathological conditions, including cancer, neurodegenerative diseases, and ischemia–reperfusion injury [1,2,3]. Distinct from apoptosis, ferroptosis occurs independently of caspase activation and is morphologically and biochemically characterized by shrunken mitochondria, a profound depletion of the antioxidant glutathione (GSH), and the lethal accumulation of lipid-reactive oxygen species (ROS) [4,5]. The key mechanism of ferroptosis resistance involves the synergistic action of glutathione peroxidase 4 (GPX4) and its cofactor, GSH. GPX4 utilizes GSH as a cofactor to convert cytotoxic lipid hydroperoxides into non-toxic lipid alcohols [5,6]. Maintenance of GPX4-mediated ferroptosis defense is fundamentally dependent on a continuous supply of NADPH. This essential reducing equivalent facilitates the regeneration of GSH from its oxidized form GSSG and is primarily derived from metabolic pathways including the pentose phosphate pathway (PPP), with glucose-6-phosphate dehydrogenase (G6PD) and 6-phosphogluconate dehydrogenase (6PGD) serving as the rate-limiting enzymes [7]. NADPH is also derived through the activity of malic enzyme 1 (ME1) [8,9].

The cellular pool of NADPH is inextricably linked to the activity of NAD kinase (NADK, also referred to as NADK1), which is primarily cytosolic and catalyzes the phosphorylation of NAD^+^ to NADP^+^, the direct precursor for NADPH synthesis [10,11,12]. While a mitochondrial isoform, NADK2, converts NADH to NADPH to support proline biosynthesis, its contribution to the overall cytosolic NADPH pool is limited [13,14]. Thus, cytosolic NADK serves as a master regulator of redox homeostasis by controlling the substrate availability for NADPH-producing enzymes.

Although NADPH availability is well established as a critical determinant of ferroptosis sensitivity [12,15], and NADK has been implicated in this process, the precise mechanistic role of NADK remains incompletely defined. For example, while supplementation with the NAD^+^ precursor nicotinamide mononucleotide (NMN) has been shown to protect against ferroptosis [16], the extent to which this effect depends on NADK-maintained NADP(H) levels remains unclear. Notably, NADP^+^ serves as an essential cofactor for a multitude of enzymes. These enzymes include G6PD, ME1, isocitrate dehydrogenase 1 (IDH1), phosphogluconate dehydrogenase (PGD), and aldehyde dehydrogenase 1 family 1 member L1 (ALDH1L1), as well as mitochondrial IDH2, ME3, ALDH1L2, and glutamate dehydrogenase 1 (GDH1), with the exception of GDH1, whose primary product is NADH. These enzymes generate NADPH by catalyzing the dehydrogenation of their respective substrates. In contrast, mitochondrial nicotinamide nucleotide transhydrogenase (NNT) produces NADPH through transhydrogenation using NADH [17]. Although these NADP^+^-dependent enzymes are known to participate in ferroptosis regulation [7,9,18,19,20,21,22,23], their functional reliance on NADK-derived NADP^+^ has not been systematically investigated. This proposed mechanism currently lacks experimental validation, and the absence of such knowledge hinders a deeper understanding of the metabolic hierarchy within the ferroptosis defense network.

To address these pivotal questions, our study employs a multi-faceted strategy, integrating pharmacological inhibition, genetic silencing, and detailed metabolic analyses to precisely delineate the role of NADK in ferroptosis. We demonstrate that NADK is a fundamental governor of ferroptosis susceptibility, primarily through its role in maintaining the intracellular NADPH reservoir, which in turn sustains GSH homeostasis and GPX4 activity. Furthermore, we identify NADK as an essential metabolic nexus that enables the function of several known ferroptosis rescue pathways. Our data establish that the protective effects of NMN, as well as the anti-ferroptosis activities of G6PD and ME1, are contingent upon NADK, thereby positioning it upstream of these key factors. These findings not only elucidate NADK as a central metabolic switch controlling ferroptosis but also highlight its significant potential as a therapeutic target for modulating ferroptosis in relevant disease contexts.

## 2. Materials and Methods

### 2.1. Cell Culture and Reagents

HT1080 cells, (American Type Culture Collection, ATCC accession number: CCL-121), kindly provided by Dr. Xuexian Fang, were cultured in Dulbecco’s Modified Eagle Medium (DMEM) supplemented with 10% (*v*/*v*) fetal bovine serum (FBS), 2 mM L-glutamine, 1 mM sodium pyruvate, 100 U/mL penicillin, and 100 µg/mL streptomycin under standard cell culture conditions (37 °C, 5% CO_2_). HT1080 cells, originally isolated from the connective tissue of a 35-year-old male Caucasian patient with fibrosarcoma, are well-established to harbor an activated N-ras oncogene. These cells are frequently employed in ferroptosis research due to their documented sensitivity to ferroptosis inducers such as RSL3. To ensure experimental reproducibility, HT1080 cells within passages 3 to 6 were used in all experiments.

Ferroptosis was induced using the specific ferroptosis inducers RSL-3 (Cat# GC12431), iFSP1(Cat# GC45928) or BQR (Brequinar; Cat# GC19082) (GlpBio, Montclair, CA, USA). The following compounds were utilized: nicotinamide mononucleotide (NMN, GlpBio; Cat# GC16971), thionicotinamide (thioNAM, GlpBio Cat# GD22321), and Ferrostatin-1 (TargetMol, Shanghai, China; Cat# 347174-05-4).

### 2.2. Cell Viability and Cytotoxicity Assay

Cell viability was assessed using a CCK-8 kit (Beyotime, Shanghai, China) in accordance with the manufacturer’s instructions. Briefly, cells were plated in 96-well plates at a density of 5 × 10^3^ cells per well and exposed to the designated experimental conditions. Following treatment, the cells were incubated with the CCK-8 reagent for 2 h at 37 °C, after which the absorbance was quantified at 450 nm using a SPARK multimode microplate reader (Tecan, Männedorf, Switzerland). All assays were conducted with three technical replicates per condition and repeated in three independent biological replicates.

### 2.3. RNA Interference (RNAi)-Mediated Gene Silencing

Gene silencing was achieved via transient transfection using Lipofectamine 3000 transfection reagent (Invitrogen, Carlsbad, CA, USA) with NADK-specific small interfering RNA (siRNA; 5′-GGA GAA CAT GAT CGT GTA T-3′) or a non-targeting scrambled control siRNA (Quanyang Biotechnology, Shanghai, China). The efficacy of NADK knockdown was systematically evaluated at both the transcriptional (mRNA) and translational (protein) levels 24 h post-transfection.

### 2.4. Quantitative Real-Time Polymerase Chain Reaction (qRT-PCR)

Total RNA was isolated using TRIzol reagent (Invitrogen, Carlsbad, CA, USA) and subsequently reverse-transcribed employing the Thermoscript™ RT-PCR System (Thermo Fisher Scientific, Waltham, MA, USA). Quantitative PCR amplification was conducted on a QuantStudio 6 Flex Real-Time PCR System (Applied Biosystems, Waltham, MA, USA) utilizing SYBR Green Master Mix (Takara Bio Inc., Otsu, Japan). The sequences of all primers utilized in this study are presented in Table 1. Relative quantification of mRNA expression levels was performed using the 2^−ΔΔCt^ method, with β-actin serving as the internal reference gene for normalization.

### 2.5. Western Blot Analysis

Cell lysates were prepared by homogenizing cells in RIPA lysis buffer containing a cocktail of protease inhibitors (Beyotime). The total protein concentration was determined using the bicinchoninic acid (BCA) assay. Equivalent amounts of protein (20 μg per lane) were resolved by 10% sodium dodecyl sulfate-polyacrylamide gel electrophoresis (SDS-PAGE) and subsequently electrotransferred onto polyvinylidene difluoride (PVDF) membranes. Membranes were blocked with 5% (*w*/*v*) non-fat dry milk to prevent nonspecific binding, followed by overnight incubation at 4 °C with the following primary antibodies: anti-ME1 (1:1000, ABclonal, Wuhan, China), anti-GPX4 (1:2000, ABclonal), anti-NADK (1:1000, Proteintech, Wuhan, China), anti-G6PD (1:500, Cloud-Clone Corp, Wuhan, China), along with loading control antibodies against β-Actin or GAPDH (1:5000, Abcam, Cambridge, MA, USA). Immunoreactive bands were detected using an enhanced chemiluminescence (ECL) substrate (Fdbio Science, Hangzhou, China) and quantified via densitometric analysis using ImageJ software (version 2.1.4.7; National Institutes of Health, Bethesda, MD, USA).

### 2.6. NAD(H)/NADP(H) Quantification

The cellular levels of NAD(H) and NADP(H) were quantified using the NAD^+^/NADH Assay Kit and NADP^+^/NADPH Assay Kit, respectively, following the manufacturer’s protocols (Shanghai Beyotime) as described previously [24]. Briefly, cells were seeded in six-well plates at a density of 3 × 10^5^ cells per well and incubated overnight. After experimental treatments, cells were washed three times with ice-cold phosphate-buffered saline (PBS), lysed with 200 µL of either NADP^+^/NADPH or NAD^+^/NADH extraction buffer, and centrifuged to collect the supernatant as the assay sample.

For quantification, 20 µL or 50 µL of the lysate was transferred to a 96-well microplate, mixed with the corresponding enzyme working solution (alcohol dehydrogenase for NAD^+^/NADH or glucose-6-phosphate dehydrogenase (G6PD) for NADP^+^/NADPH), and incubated at 37 °C for 10 min. Subsequently, the colorimetric reagent WST-8 (provided in the kit) was added to generate formazan, and the reaction was further incubated at 37 °C for 30 min. The orange product (formazan) was then measured at 450 nm spectrophotometrically. Total concentrations of NAD^+^/NADP^+^ were calculated based on a standard curve. All assays were performed in triplicate and repeated in at least three independent experiments.

### 2.7. Determination of Malondialdehyde (MDA) Level

The MDA level was quantified following the manufacturer’s protocol provided in the MDA assay kit (Shanghai Beyotime). Treated cells were lysed using RIPA buffer, and the resulting cell lysates were resuspended in 100 µL of phosphate-buffered saline (PBS) to prepare the test samples. For each sample, 70 µL of the prepared cell lysate was combined with 200 µL of the MDA working solution in an Eppendorf (EP) tube. The mixture was thoroughly mixed, sealed, and incubated in a boiling water bath (100 °C) for 15 min to facilitate the MDA-thiobarbituric acid (TBA) reaction. Following incubation, the reaction mixture was centrifuged at 1000× *g* for 10 min to pellet precipitated proteins. Subsequently, 200 µL of the supernatant was carefully transferred to a 96-well microplate, and the absorbance was measured at 532 nm using a microplate reader. The MDA concentration in each sample was determined by referencing a standard curve generated with known MDA concentrations. All assays were performed in triplicate, and experiments were independently repeated at least three times to ensure reproducibility.

### 2.8. Determination of GSH Level

The GSH levels were quantified using the GSH and GSSG Detection Kit (Shanghai Beyotime) following the manufacturer’s protocol as described previously [24]. After trypsinization and cell harvesting, 30 μL of protein removal reagent M (provided in the kit and prepared immediately before use) was added to resuspend the cell pellet. The mixture was vigorously vortexed and subsequently lysed by rapid agitation in liquid nitrogen or incubation in a 37 °C water bath. The resulting lysate was centrifuged at 10,000× *g* for 10 min at 4 °C, and the supernatant was collected as the test sample.

For the assay, 10 μL of the test sample and 10 μL of the standard solution were pipetted into individual wells of a 96-well plate. Subsequently, 150 μL of the total glutathione detection working solution was added, followed by thorough mixing. The plate was incubated for 5 min at room temperature, after which 50 μL of 0.5 mg/mL NADPH was introduced, mixed, and the absorbance at 412 nm was measured using a microplate reader. The total GSH + GSSG concentration was determined based on the standard curve.

To measure GSSG content, 10 μL of the test sample and 10 μL of the standard were added to a new 96-well plate, followed by 2 μL of GSH scavenging auxiliary solution, which was vortexed thoroughly. Then, 0.4 μL of GSH scavenging reagent working solution was added, mixed, and the mixture was incubated at 25 °C for 60 min. The total GSH + GSSG measurement procedure was repeated, and the cellular GSSG content was calculated. The GSH concentration was derived using the formula provided in the kit. The GSH/GSSG ratio was computed and used as the final result. All samples were assayed in at least three technical replicates.

Reduced glutathione (GSH) and oxidized glutathione (GSSG) were quantified via a colorimetric assay (Beyotime) employing the glutathione reductase recycling method. All measurements were performed in triplicate with three independent biological replicates.

### 2.9. Determination of Reactive Oxygen Species (ROS) Level

Intracellular ROS levels were measured using the 2′,7′-dichlorodihydrofluorescein diacetate (DCFH-DA) fluorescent probe (Beyotime). Following treatment, cells were incubated with 10 μM DCFH-DA in serum-free culture medium for 20 min at 37 °C. Subsequently, the cells were washed with phosphate-buffered saline (PBS) and analyzed using a NovoCyte Quanteo flow cytometer (Agilent Technologies, Santa Clara, CA, USA) equipped with 488 nm excitation and 530 nm emission filters. Flow cytometric data were processed and analyzed using FlowJo software (version 10.8.1, BD Biosciences, BD Biosciences, Franklin Lakes, NJ, USA).

### 2.10. Statistical Analysis

All quantitative data are expressed as the mean ± standard deviation (SD) derived from a minimum of three independent biological replicates. Statistical analyses were performed using GraphPad Prism 9.0 software, employing either an unpaired two-tailed Student’s *t*-test for pairwise comparisons or one-way analysis of variance (ANOVA) followed by Tukey’s multiple comparison post-hoc test for group-wise assessments, and two-way ANOVA for comparisons involving two factors within groups. Differences were considered statistically significant when *p* < 0.05.

## 3. Results

### 3.1. Pharmacological Inhibition of NADK Sensitizes Cells to Ferroptosis

**Figure 1:**
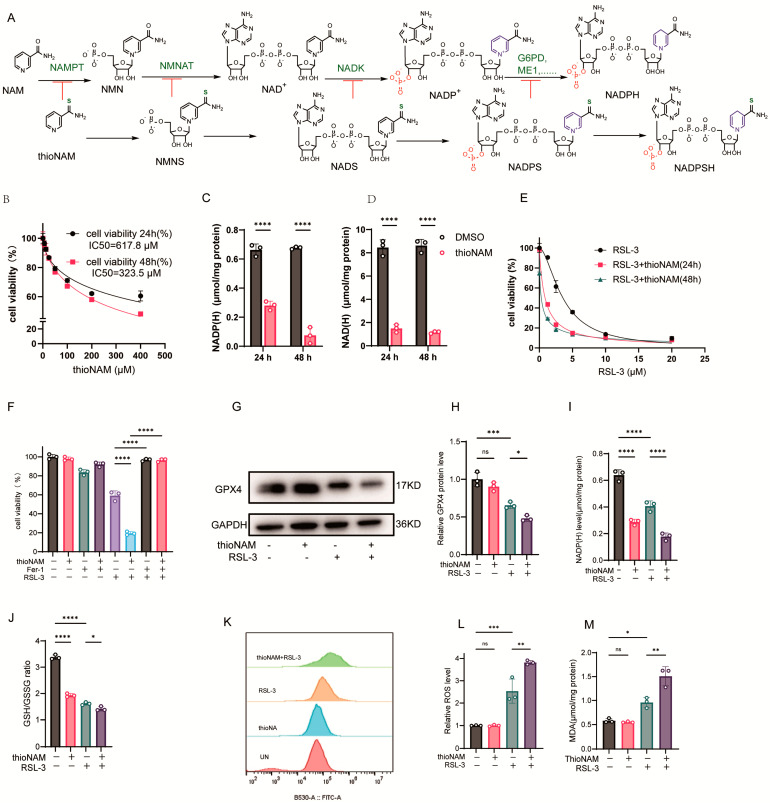
ThioNAM Depletes NADP(H) and Sensitizes Cells to Ferroptosis (**A**) Schematic diagram of thioNAM and its metabolites inhibiting enzymes involved in NADPH generation. (**B**) Cell viability assessment via CCK-8 assay following 24 h or 48 h treatment with varying thioNAM concentrations. (**C**,**D**) Quantification of (**C**) NADP(H) and (**D**) NAD(H) levels in cells treated with 50 μM thioNAM for 24 h or 48 h. (**E**) Cell viability assessment via CCK-8 assay in cells pretreated with 50 μM thioNAM for 24 h or 48 h, followed by 24 h exposure to gradient RSL-3 concentrations. (**F**) Cell viability analysis employing CCK-8 assay in cells preincubated with or without 50 μM thioNAM for 24 h, subsequently treated with 2.5 μM RSL-3 and 10 μM Fer-1 for an additional 24 h. (**G**–**M**) Cells were pretreated with 50 μM thioNAM for 24 h, then co-treated with 2.5 μM RSL-3 for an additional 24 h, (**G**) GPX4 protein expression was analyzed by Western blot. (**H**) Densitometric quantification of GPX4 protein expression. (**I**) NADP(H) levels. (**J**) GSH/GSSG ratio. (**K**) ROS levels measured by flow cytometry. (**L**) Quantification of ROS. (**M**) Malondialdehyde (MDA) content determination. All data are presented as mean ± SD from three independent experiments. Statistical significance was determined by one-way ANOVA; ns: not significant, * *p* < 0.05, ** *p* < 0.01, *** *p* < 0.001, **** *p* < 0.0001.

Thionicotinamide (thioNAM) is an analog of nicotinamide (NAM). NAM is catalyzed by intracellular enzyme systems to generate NMN, NAD^+^, and NADP^+^, while thioNAM is similarly catalyzed to produce the corresponding thio-analogs NMNS, NADS, and NADPS. NADPS can also serve as hydrogen receptor to form NADPSH in certain dehydrogenase reactions, such as catalyzed by G6PD [25,26], but not by ME1 [27]. ThioNAM and its intracellularly generated thio-derivatives act as competitive inhibitors of Nicotinamide Phosphoribosyltransferase (NAMPT), Nicotinamide Nucleotide Adenylyltransferase 1 (NMNAT), NADK, and NADP-dependent dehydrogenases (G6PD, ME1, etc.), thereby inhibiting multiple enzymes involved in the generation of NADPH [25,28] (Figure 1A). ThioNAM reduced cell viability in a dose- and time-dependent manner, with IC_50_ values of 617.8 μM at 24 h and 323.5 μM at 48 h, respectively. Treatment with 50 μM thioNAM for 24 h or 48 h only slightly reduces the cell viability (79% at 24 h and 76% at 48 h; Figure 1B). After 24 h and 48 h of treatment with 50 μM thioNAM, intracellular NADPH levels decreased by 53% and 89%, respectively (Figure 1C), while NADH levels also decreased by 83% and 87%, respectively (Figure 1D). Pre-treatment with 50 μM thioNAM for 24 h and 48 h significantly enhanced cellular sensitivity to RSL-3-induced ferroptosis (Figure 1E). Therefore, a concentration of 50 μM was selected, as it enabled the assessment of NADPH depletion effects under conditions that minimized direct cytotoxicity. We also evaluated the effect of thioNAM on ferroptosis induced by other ferroptosis inducers, iFSP1 and BQR. The results showed that only pretreatment with thioNAM for 48 h, but not for 24 h, sensitized HT1080 cells to iFSP1. In contrast, thioNAM pretreatment did not affect BQR-induced ferroptosis (Supplementary Figure S1). The thioNAM-enhanced cell death could be completely rescued by the ferroptosis inhibitor ferrostatin-1 (Fer-1) (Figure 1F).

Mechanistically, ThioNAM treatment alone did not exert a significant effect on GPX4 protein expression; however, it potentiated the capacity of RSL-3 to downregulate GPX4 protein levels (Figure 1G, H). ThioNAM synergized with RSL-3 to downregulate intracellular NADP(H) levels (Figure 1I) and reduce the GSH/GSSG ratio (Figure 1J). Although thioNAM treatment alone did not increase basal ROS and MDA levels, it potentiated RSL-3-induced oxidative stress (Figure 1K–M). These results indicate that thioNAM promotes ferroptosis by inhibiting NADK and reducing NADP(H) and (or) NAD(H) levels.

### 3.2. Genetic Knockdown of NADK Amplifies Ferroptosis

**Figure 2:**
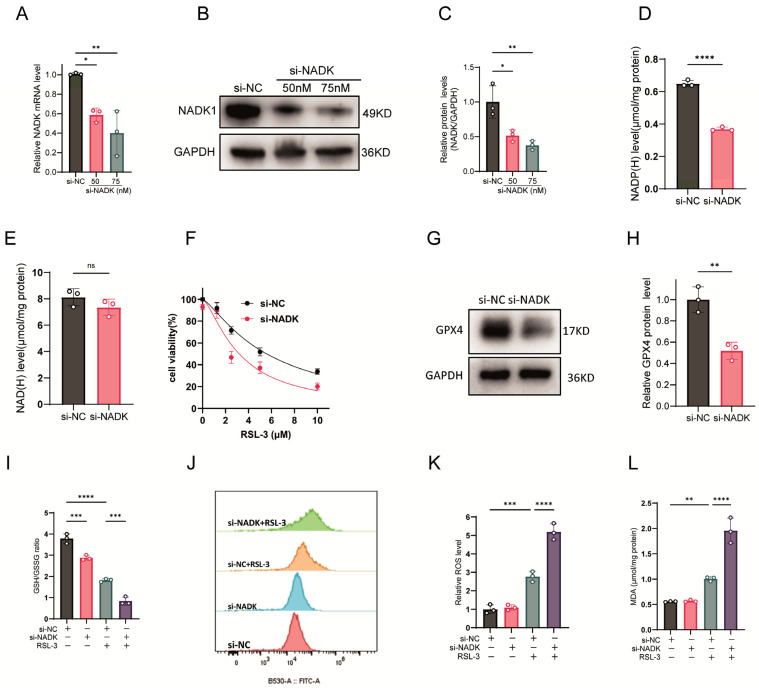
NADK Knockdown Exacerbates Ferroptosis. (**A**–**C**) HT1080 cells were transfected with serial concentrations of NADK-specific small interfering RNA (si-NADK) or non-targeting scrambled siRNA (si-NC), with cells harvested 24 h post-transfection. (**A**) NADK transcript levels were quantified by qRT-PCR. (**B**) NADK protein abundance was examined by Western blot analysis. (**C**) Densitometric quantification of NADK protein bands was conducted. (**D**,**E**) Following 24-h transfection with 75 nM si-NADK, cellular (**D**) NADP(H) and (**E**) NAD(H) levels were measured. (**F**) Cell viability was evaluated in response to increasing concentrations of the ferroptosis inducer RSL-3 (24-h treatment) via CCK-8 assay. (**G**,**H**) GPX4 protein expression and corresponding band densitometry were assessed by Western blot in cells treated with 75 nM si-NADK for 24 h. (**I**–**L**) HT1080 cells were transfected with 75 nM si-NADK for 24 h, followed by treatment with 2.5 μM RSL-3 for an additional 24 h prior to sample collection for subsequent analyses: (**I**) GSH/GSSG ratio; (**J**) ROS levels measured by flow cytometry; (**K**) Quantification of ROS. (**L**) MDA levels. All data are expressed as mean ± standard deviation (SD) from three biologically independent replicates. Statistical significance was determined by one-way ANOVA with post-hoc analysis; ns: not significant, * *p* < 0.05, ** *p* < 0.01, *** *p* < 0.001, **** *p* < 0.0001.

ThioNAM and its metabolites act as competitive inhibitors of multiple enzymes involved in NADP(H) and NAD(H) generation, including NADK. To further investigate the specific role of NADK in ferroptosis, we employed RNA interference to knock down NADK expression. siRNA-mediated NADK knockdown (75 nM, 24 h) decreased both mRNA and protein levels (Figure 2A–C). Unlike thioNAM, siRNA targeting NADK selectively decreased NADP(H) levels (Figure 2D) without affecting NAD(H) levels (Figure 2E). Knockdown of NADK increased cellular sensitivity to RSL-3-induced ferroptosis (Figure 2F).

Knockdown of NADK downregulated GPX4 protein expression (Figure 2G,H), further reduced NADP(H) levels in RSL-3-treated cells (Figure 2I), and decreased the GSH/GSSG ratio (Figure 2J). Although knockdown of NADK alone did not increase intracellular ROS or MDA levels, it significantly enhanced RSL-3-induced ROS and MDA production (Figure 2K–L).

### 3.3. Overexpression of NADK Restores NADP(H) and Confers Ferroptosis Resistance

**Figure 3:**
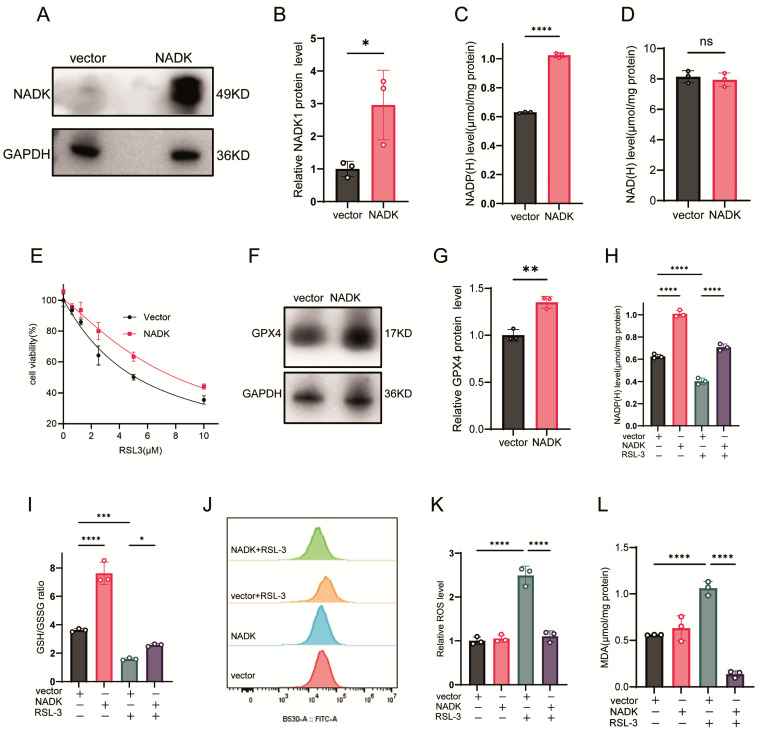
NADK Overexpression Restores Redox Homeostasis and Rescues Ferroptosis. (**A**–**D**) HT1080 cells were transfected with 2.5 μg of NADK plasmid or empty vector (vector) for 24 h: (**A**) NADK protein levels detected by Western blot; (**B**) Quantification of NADK protein expression; (**C**) NADP(H) levels; (**D**) NAD(H) levels. (**E**) Cells were transfected with 2.5 μg NADK plasmid for 24 h before being treated with different concentrations of RSL-3 for another 24 h, and cell viability was measured by CCK-8 assay. (**F**,**G**) Cells transfected with 2.5 μg NADK plasmid for 24 h: (**F**) GPX4 protein expression detected by Western blot. (**G**) Quantification of GPX4 protein expression. (**H**–**L**) HT1080 cells were transfected with 2.5 μg NADK plasmid for 24 h, then treated with 2.5 μM RSL-3: (**H**) NADP(H) levels; (**I**) GSH/GSSG ratio; (**J**) ROS levels measured by flow cytometry; (**K**) Quantification of ROS by flow cytometry; (**L**) MDA levels. All data are presented as mean ± SD from three independent biological replicates. Statistical significance was determined by one-way ANOVA; * *p* < 0.05, ** *p* < 0.01, *** *p* < 0.001, **** *p* < 0.0001; ns, not significant.

We further investigated the effect of NADK overexpression on NADP(H) levels and ferroptosis sensitivity. The expression of NADK protein was confirmed by western-blot assay (Figure 3A,B). NADK increased intracellular NADP(H) levels (Figure 3C) but NAD(H) levels were not affected (Figure 3D). It also attenuated RSL-3 induced ferroptosis (Figure 3E). Additionally, NADK upregulated GPX4 protein expression (Figure 3F,G), restored NADP(H) levels in RSL-3-treated cells (Figure 3H), and elevated the GSH/GSSG ratio (Figure 3I). While NADK overexpression alone did not affect basal ROS or MDA levels, it significantly reduced ROS and MDA levels in cells treated with RSL-3 (Figure 3J–L).

### 3.4. NADK Is Required for G6PD-Mediated Ferroptosis Resistance

**Figure 4:**
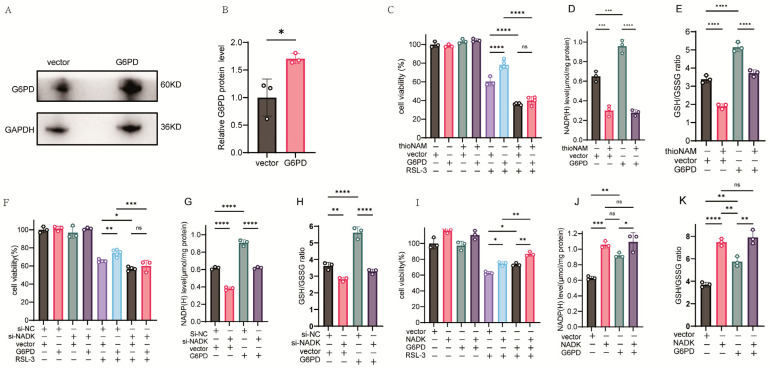
NADK Activity Is Required for G6PD-Mediated Ferroptosis Resistance. (**A**,**B**) HT1080 cells were transfected with 2.5 μg of G6PD expression plasmid or empty vector control for 24 h. (**A**) G6PD protein expression was analyzed by Western blot analysis. (**B**) Quantitative analysis of G6PD protein levels normalized to loading controls. (**C**) Cell viability was assessed by CCK-8 assay following pretreatment with thioNAM, transfection with 2.5 μg G6PD plasmid for 24 h, and subsequent treatment with 2.5 μM RSL-3 for an additional 24 h. (**D**,**E**) Cells were pretreated with thioNAM, transfected with 2.5 μg G6PD plasmid for 24 h, and then harvested for: (**D**) Quantification of cellular NADP(H) levels. (**E**) Measurement of GSH concentrations. (**F**) Cell viability was determined by CCK-8 assay following co-transfection with 2.5 μg G6PD plasmid and 75 nM NADK-specific siRNA (si-NADK) for 24 h, followed by treatment with 2.5 μM RSL-3 for an additional 24 h. (**G**,**H**) HT1080 cells were co-transfected with 2.5 μg G6PD plasmid and 75 nM si-NADK for 24 h, then collected for: (**G**) Quantification of NADP(H) levels. (**H**) Measurement of GSH concentrations. (**I**) Cell viability was determined by CCK-8 assay following co-transfection with 2.5 μg G6PD plasmid and 2.5 μg NADK expression plasmid for 24 h, followed by treatment with 2.5 μM RSL-3 for 24 h. (**J**,**K**) HT1080 cells were co-transfected with 2.5 μg G6PD plasmid and 2.5 μg NADK plasmid for 24 h, then harvested for: (**J**) Quantification of NADP(H) levels; (**K**) Measurement of GSH concentrations. All data are presented as mean ± standard deviation (SD) from three independent biological replicates. Statistical significance was determined by one-way ANOVA; ns: not significant, * *p* < 0.05, ** *p* < 0.01, *** *p* < 0.001, **** *p* < 0.0001.

HT1080 cells were transfected with a G6PD plasmid and the expression of the corresponding protein was confirmed by western-blot assay (Figure 4A,B). Overexpression of G6PD enhanced cellular resistance to ferroptosis; however, this effect was abolished upon concurrent treatment with thioNAM (Figure 4C). G6PD overexpression elevated intracellular NADP(H) levels, an effect that was also abolished by thioNAM (Figure 4D). Furthermore, G6PD elevated cellular GSH content and the GSH/GSSG ratio (Figure 4E), and this function was impaired by thioNAM. Similarly, knockdown of NADK abrogated the protective effect of G6PD against ferroptosis (Figure 4F), attenuated the G6PD-mediated increase in NADP(H) (Figure 4G), and reduced its ability to regulate the GSH/GSSG ratio (Figure 4H).

Conversely, overexpression of NADK enhanced the anti-ferroptosis function of G6PD (Figure 4I). Overexpression of both G6PD and NADK led to an increase in NADP(H) levels, with NADK demonstrating a more pronounced effect, although this difference was not statistically significant. Furthermore, the co-expression of G6PD and NADK did not yield NADP(H) levels significantly higher than those observed in cells expressing NADK alone. (Figure 4J). Both NADK and G6PD increase the GSH/GSSG ratio, with NADK demonstrating a more pronounced effect. However, co-transfection of NADK and G6PD did not result in a significant further increase in the GSH/GSSG ratio compared to transfection with NADK alone. (Figure 4K).

### 3.5. NADK Is Required for ME1-Mediated Ferroptosis Resistance

**Figure 5:**
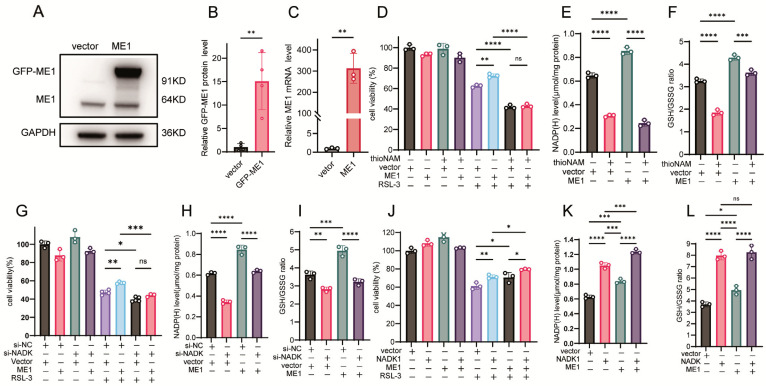
NADK Governs the Ferroptosis-Rescuing Function of ME1. (**A**–**C**) HT1080 cells were transfected with 2.5 μg of ME1 plasmid or empty vector for 24 h: (**A**) ME1 protein levels detected by Western blot; (**B**) Quantification of ME1 protein expression; (**C**) ME1 mRNA levels measured by qPCR. (**D**) Cell viability was assessed by CCK-8 assay following pretreatment with thioNAM, transfection with 2.5 μg ME1 plasmid for 24 h, and subsequent treatment with 2.5 μM RSL-3 for the following 24 h. (**E**,**F**) Cells were pretreated with thioNAM, transfected with 2.5 μg ME1 plasmid for 24 h, and then harvested for: (**E**) NADP(H) quantification; (**F**) Measurement of GSH concentrations. (**G**) Cells were co-transfected with 2.5 μg ME1 plasmid and 75 nM si-NADK for 24 h, followed by treatment with 2.5 μM RSL-3 for an additional 24 h. Cell viability was determined by CCK-8 assay. (**H**,**I**) HT1080 cells were co-transfected with 2.5 μg ME1 plasmid and 75 nM si-NADK for 24 h, then collected for: (**H**) NADP(H) measurement. (**I**) Measurement of GSH concentrations. (**J**) Cells were co-transfected with 2.5 μg ME1 plasmid and 2.5 μg NADK plasmid for 24 h, followed by treatment with 2.5 μM RSL-3 for 24 h. Cell viability was measured by CCK-8 assay. (**K**,**L**) HT1080 cells were co-transfected with 2.5 μg ME1 plasmid and 2.5 μg NADK plasmid for 24 h, then harvested for: (**K**) NADP(H) quantification. (**L**) Measurement of GSH concentrations. Data are presented as mean ± SD from three independent experiments. *p*-values were determined using ANOVA; ns: not significant, * *p* < 0.05, ** *p* < 0.01, *** *p* < 0.001, **** *p* < 0.0001.

ME1 catalyzes the malate dehydrogenation and decarboxylation reaction coupled with NADPH generation, serving as another key pathway for maintaining intracellular NADPH homeostasis. Recent studies have further confirmed its role in countering ferroptosis [9]. We next examined whether NADK is involved in regulating ME1-mediated effects on ferroptosis. Transfection with a GFP-ME1 expression vector resulted in increased GFP-ME1 protein and ME1 mRNA levels (Figure 5A–C). Overexpression of ME1 conferred resistance to ferroptosis and elevated NADPH levels; however, these effects were abolished by thioNAM treatment (Figure 5D,E). ThioNA also attenuated the ability of ME1 to increase the GSH/GSSG ratio (Figure 5F). Knockdown of NADK eliminated the protective effect of ME1 against ferroptosis and impaired its capacity to enhance NADPH levels and the GSH/GSSG ratio (Figure 5G–I). Cells co-transfected with NADK and ME1 exhibited stronger resistance to ferroptosis than those expressing ME1 alone (Figure 5J). Furthermore, NADK overexpression demonstrated a more pronounced effect than ME1 in elevating intracellular NADPH levels and the GSH/GSSG ratio (Figure 5K,L).

### 3.6. NADK Is Required for NMN-Mediated Ferroptosis Resistance

**Figure 6:**
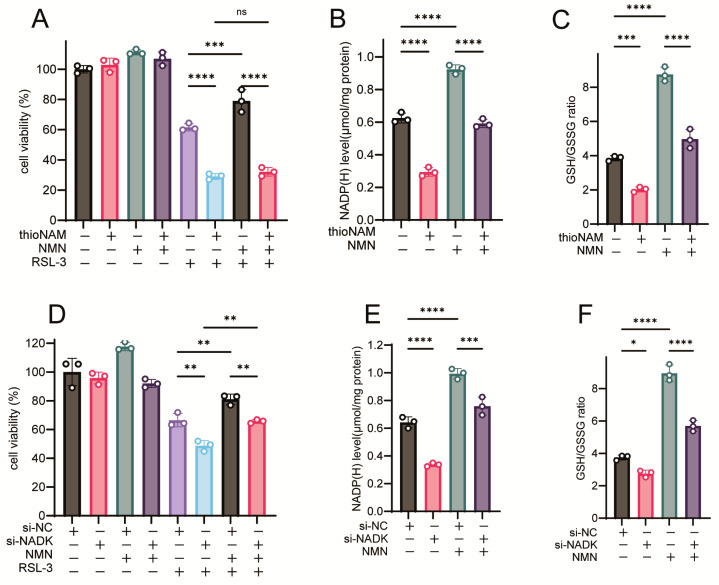

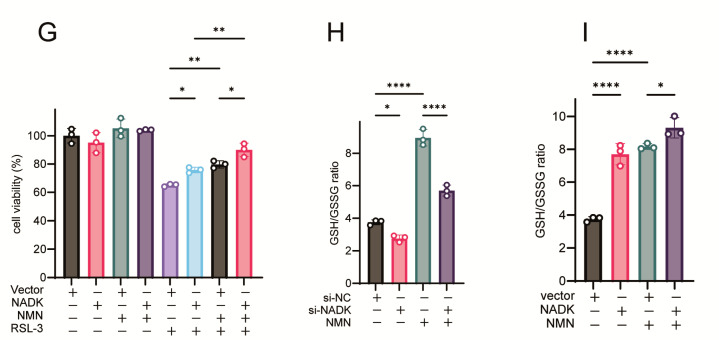
NADK Controls the Function of NMN in Rescuing Ferroptosis. (**A**) Cells were pretreated with 50 μM thioNAM for 24 h, followed by co-treatment with 2.5 μM RSL-3 and 1 mM NMN for 24 h. Cell viability was measured by CCK8 assay. (**B**,**C**) Cells were pretreated with 50 μM thioNAM for 24 h, then treated with 1 mM NMN for 2 h. Cells were collected to measure (**B**) NADP(H) levels and (**C**) GSH levels. (**D**) Cells were transfected with 75 nM Si-NADK for 24 h, followed by co-treatment with 2.5 μM RSL-3 and 1 mM NMN for 24 h. Cell viability was measured by CCK8 assay. (**E**,**F**) Cells were transfected with Si-NADK for 24 h, then treated with 1 mM NMN for 2 h. Cells were collected to measure (**E**) NADP(H) levels and (**F**) GSH levels. (**G**) Cells were transfected with a 2.5 μg NADK plasmid, followed by co-treatment with 2.5 μM RSL-3 and 1 mM NMN for 24 h. Cell viability was measured by CCK8 assay. (**H**,**I**) Cells were transfected with a 2.5 μg NADK plasmid, then treated with 1 mM NMN for 2 h. Cells were collected to measure (**H**) NADP(H) levels and (**I**) GSH levels. Results are presented as mean ± standard deviation from three independent experiments. *p* values were determined using one-way ANOVA. ns, not significant; * *p* < 0.05, ** *p* < 0.01, *** *p* < 0.001, **** *p* < 0.0001.

The ferroptosis-rescuing efficacy of NMN was abrogated by thioNAM (Figure 6A). While NMN treatment increased intracellular NADP(H) concentrations, thioNAM significantly attenuated this NMN-induced elevation (Figure 6B). Concomitantly, thioNAM also mitigated the NMN-mediated augmentation of the GSH/GSSG ratio (Figure 6C). Genetic silencing of NADK via siRNA-mediated knockdown impaired the ferroptosis-protective effects of NMN (Figure 6D) and concurrently diminished NMN-induced modulation of NADP(H) content and the GSH/GSSG ratio (Figure 6E,F). Conversely, NADK overexpression potentiated the ferroptosis-rescuing activity of NMN (Figure 6G), further amplified NMN-induced NADP(H) accumulation (Figure 6H), and synergistically increased the GSH/GSSG ratio (Figure 6I).

## 4. Discussion

Mammalian cells depend on the interconversion of oxidized NADP^+^ and reduced NADPH forms to support reductive biosynthesis and to maintain cellular antioxidant defense. Notably, NADP(H) exhibits limited membrane permeability and is largely confined to specific subcellular compartments [29]. Mitochondrial-localized NADK2 predominantly facilitates proline biosynthetic processes in cultured cells [13,14]. However in vivo mitochondrial NADPH is also important for mitochondrial fatty acid and lipoic acid synthesis, and to fuel the reductases TRXR2 and GSR that are essential for H_2_O_2_ and phospholipid and fatty acid hydroperoxide detoxification [30,31]. In contrast, cytosolic-localized NADK emerges as the primary mediator of NADP(H) biosynthesis across most mammalian cell types [32]. Human MESH1 is a cytosolic NADPH phosphatase that catalyzes the hydrolysis of the 2′-phosphate group of NADPH to produce NADH and inorganic phosphate. It has been shown to sensitize cells to ferroptosis by depleting NADPH levels [33,34]. NADPH abundance serves as a biomarker for predicting sensitivity to ferroptosis-inducing compounds [15].

FSP1 (Ferroptosis Suppressor Protein 1) utilizes reduced coenzyme Q10 (CoQ-H_2_) as a lipophilic radical-trapping antioxidant that can halt the chain reaction of lipid peroxidation [35,36], with NADPH serving as the primary source for regenerating reduced CoQ-H_2_ [37]. DHODH is localized in both mitochondria and the cytoplasm, and its inhibitor is BQR. Mitochondrial DHODH has been reported to participate in the defense mechanism against ferroptosis [38]. In our experiments, HT1080 cells were most sensitive to RSL-3, a GPX4 inhibitor that relies on the NADPH/GSH system. They showed intermediate sensitivity to iFSP1, an FSP1 inhibitor partially dependent on NADPH for CoQH_2_ regeneration, which was also influenced by thioNAM pretreatment. In contrast, cells were resistant to BQR, a DHODH inhibitor independent of cytosolic NADPH, whose ferroptosis induction remained unaffected by thioNAM. These findings indicate that the sensitizing effect of thioNAM on ferroptosis depends on the type of inducer used, further supporting the conclusion that NADK specifically regulates ferroptosis by controlling cellular NADP(H) availability. Here, we elucidate the pivotal role of NADK in ferroptosis regulation through its critical function as a metabolic bridge linking NAD^+^ metabolism to NADPH-dependent antioxidant defense mechanisms.

Our findings demonstrate that NADK serves as a central metabolic regulator by catalyzing the conversion of NMN-derived NAD^+^ to NADP^+^, which is subsequently reduced to NADPH via the enzymatic activities of G6PD, ME1 and other dehydrogenases. This metabolic cascade sustains GSH levels and maintains GPX4 activity, thereby effectively scavenging lipid peroxides and conferring protection against ferroptosis.

Our experimental data reveal that either pharmacological inhibition or genetic knockdown of NADK results in significant depletion of the NADP(H) pool, subsequently compromising the GSH-GPX4 axis. While consistent with established literature demonstrating an inverse correlation between cellular NADPH levels and ferroptosis susceptibility [15], our study uniquely identifies NADK as the rate-limiting enzymatic node governing this critical relationship. Mechanistically, NADK exerts regulatory effects that extend beyond mere substrate provision.

Through modulation of the NADP^+^/NAD^+^ ratio, it governs the enzymatic activity of NADP^+^-dependent dehydrogenases, including G6PD and ME1. G6PD is the rate-limiting enzyme in the pentose phosphate pathway (PPP), which utilizes NADP^+^ as an essential cofactor for NADPH generation [7]. ME1, which catalyzes the conversion of malate to pyruvate while concomitantly producing NADPH, relies on NADK-mediated cofactor pool replenishment for its functional activity [9,23]. Our experimental findings demonstrate that neither the overexpression of G6PD and ME1 nor their upregulation conferred protection against ferroptosis upon NADK inhibition. Furthermore, NADK inhibition attenuated the ability of these enzymes to increase NADP(H) levels and the GSH/GSSG ratio. In contrast, NADK overexpression enhanced the capacity of G6PD and ME1 to rescue cells from ferroptosis, as well as their ability to elevate NADP(H) levels and the GSH/GSSG ratio. These results highlight the critical role of NADP^+^ bioavailability as the metabolic foundation for the ferroptosis-rescuing functions of these enzymes, with NADK serving as the key regulator controlling cellular NADP^+^ levels.

NMN, a key precursor of nicotinamide adenine dinucleotide (NAD^+^), has garnered significant interest due to its demonstrated anti-aging and cytoprotective properties [39,40,41]. Recent studies suggest that NMN alleviates ferroptosis by increasing GSH levels, enhancing the NAD^+^/NADH ratio, and elevating NADPH levels [16]. However, the precise molecular mechanisms remain unclear. Our research demonstrates that the ferroptosis-rescuing function of NMN is critically dependent on the activity of NADK. Knockdown of NADK significantly decreases intracellular NADP(H) levels and the GSH/GSSG redox ratio, whereas NAD(H) levels remain unchanged. Notably, NADK knockdown exacerbates ferroptosis and markedly attenuates the protective effect of NMN against ferroptosis. Conversely, NADK overexpression does not influence NAD(H) levels but synergizes with NMN to enhance ferroptosis rescue. These findings establish that sustaining elevated NADP(H) levels is essential for NMN-mediated ferroptosis inhibition, with NAD(H) functioning as a critical substrate for NADP(H) biosynthesis.

Additionally, our experimental results demonstrate that NADK positively regulates GPX4 protein expression. Both pharmacological inhibition and genetic knockdown of NADK significantly reduced GPX4 levels, whereas its overexpression markedly increased them, revealing a consistent positive correlation. Given the central role of the NADPH/GSH/GPX4 axis in ferroptosis suppression, and the position of NADK as the key enzyme controlling NADP^+^ supply, our finding that NADK governs GPX4 abundance suggests a previously underappreciated layer of regulation. Elucidating the precise mechanism by which NADK controls GPX4 expression, whether through direct interaction, transcriptional regulation, or redox-mediated stabilization, represents a critical future direction that will deepen our understanding of the metabolic control over ferroptosis.

These findings indicate that, beyond its role in modulating NADP+ production, NADK also coordinates NADPH-GSH homeostasis and GPX4 activity by directly controlling GPX4 expression, thereby exerting a synergistic inhibitory effect on ferroptosis.

Many cancers exhibit elevated NADK expression and demonstrate resistance to ferroptosis-inducing therapies [28,42]. Conversely, ferroptosis plays a pivotal role in the pathogenesis of neurodegenerative disorders and ischemia–reperfusion injury [9,43]. The present study suggests that NADK inhibitors (e.g., thioNAM) hold clinical potential in sensitizing tumors to ferroptosis-mediated cell death. Simultaneously, NADK activation may enhance the anti-aging effects of nutritional interventions such as NMN and improve their therapeutic efficacy in neurodegenerative disease management.

Limitations of this study include several important considerations that temper the interpretation and generalizability of the findings. First, the exclusive use of the HT1080 fibrosarcoma cell line limits tissue specificity; NADK’s role may vary across cell types due to distinct metabolism, NADK expression, and regulatory networks. Second, there is a lack of in vivo evidence; all conclusions are from in vitro experiments that do not capture whole-organism physiology, including systemic metabolism, immune interactions, and tumor microenvironment effects on NADPH pools and ferroptosis. In vivo validation in animal models or clinical samples is essential for translational relevance. Third, to further clarify the role of the NADPH/GSH/GPX4 signaling pathway in NADK-regulated ferroptosis, investigating other inducers—such as erastin, which acts upstream of GSH synthesis—would be a valuable direction for future research. Fourth, the study provides limited insight into NADK’s interplay with other ferroptosis regulators (e.g., GPX4, FSP1). Future work should broaden models, dissect interactions, and explore NADK’s interplay with other ferroptosis regulators.

## 5. Conclusions

**Figure 7:**
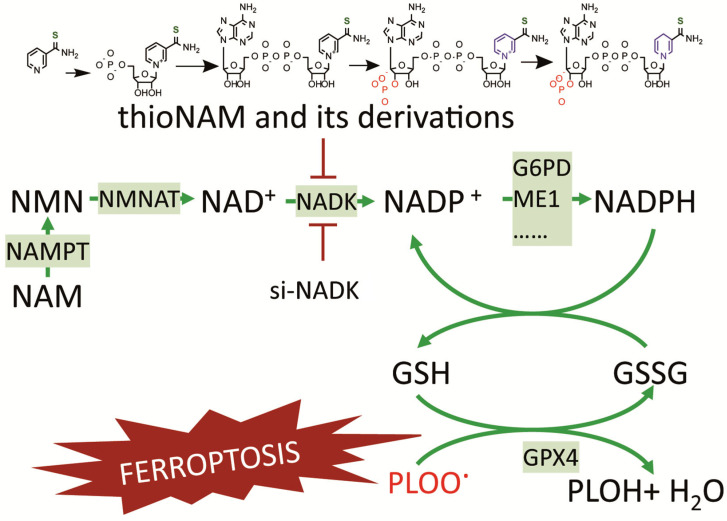
A Proposed Model for the Role of NADK in Regulating Ferroptosis through NADP^+^ Supply.

In conclusion, this study establishes cytosolic NAD kinase (NADK) as a master regulator of ferroptosis by controlling the availability of NADP^+^—the essential cofactor for NADPH-generating enzymes. NADK occupies a pivotal upstream position in the ferroptosis defense network, bridging NAD^+^ metabolism and redox homeostasis. It not only utilizes NMN-derived NAD^+^ to produce NADP^+^ but also supplies this critical cofactor to key dehydrogenases, including G6PD and ME1, thereby enabling NADPH-dependent antioxidant defense (Figure 7). Our findings demonstrate that targeting NADK disrupts this coordinated metabolic axis and sensitizes cells to ferroptosis. Therefore, NADK represents a promising therapeutic target for modulating ferroptosis in relevant diseases.

## Figures and Tables

**Table 1:** Primer sequences for quantitative real-time PCR.

Genes	Accession Numbers	Sequence (5′- 3′)
NADK	NM_001198993.2	For: GTCCTTTGATGGACGGAAGAGAC
		Rev: GAGGCTCTCAAACCAGTCGCTC
ME1	NM_002395.6	For: GAGTGCTGACATCTGACATTGA
		Rev: TTGGCTTCCGAAACACCAAAC
β-actin	NM_001101.5	For: GGGAAATCGTGCGTGACAT
		Rev: GTCAGGCAGCTCGTAGCTCTT

## Data Availability

All data that support the findings of this study are available from the corresponding author upon reasonable request.

## Supplementary Materials

The following supporting information can be downloaded at: https://www.mdpi.com/article/10.3390/antiox14121396/s1, Figure S1: Effects of ThioNAM on iFSP1- and BQR-induced ferroptosis.
