# Supplementary material for: NADK Governs Ferroptosis Susceptibility by Orchestrating NADPH Homeostasis
Source: Antioxidants (Basel). 2025 Nov 24;14(12):1396. doi: 10.3390/antiox14121396 (PMC12730079; doi:10.3390/antiox14121396)
Supplement: Supplementary file 1 [file antioxidants-14-01396-s001.zip › antioxidants-3966137-supplementary.pdf]

antioxidants

Supplementary Data

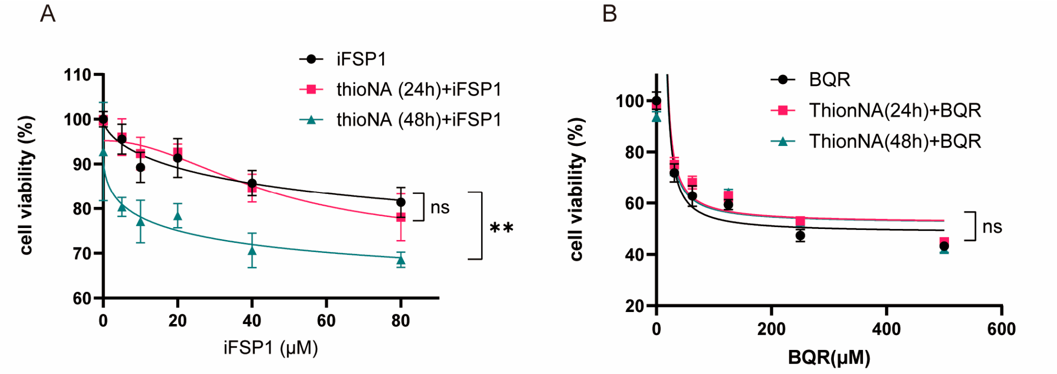

Figure S1. Effects of ThioNAM on iFSP1- and BQR-induced ferroptosis. (A) Cell viability was assessed by CCK-8 assay in cells pretreated with 50 μM ThioNAM for 24 h or 48 h, followed by exposure to increasing concentrations of iFSP1 for 24 h. (B) Cell viability was assessed by CCK-8 assay in cells pretreated with 50 μM ThioNAM for 24 h or 48 h, followed by exposure to increasing concentrations of BQR for 24 h. All data are presented as means ± SD from three independent experiments. Statistical significance was determined by one-way ANOVA; ns: not significant; ** $P < 0.01$.
